# Obstetric and offspring outcomes in isolated maternal hypothyroxinaemia: a systematic review and meta-analysis

**DOI:** 10.1007/s40618-022-01967-4

**Published:** 2022-11-23

**Authors:** L. Zhuo, Z. Wang, Y. Yang, Z. Liu, S. Wang, Y. Song

**Affiliations:** 1 Research Center of Clinical Epidemiology, Peking University Third Hospital, Beijing, People’s Republic of China; 2 Department of Endocrinology, Shandong Provincial Hospital affiliated to Shandong First Medical University, Jinan, Shandong People’s Republic of China; 3 Shandong Institute of Endocrine and Metabolic Diseases, Jinan, Shandong People’s Republic of China; 4 Department of Epidemiology and Biostatistics, School of Public Health, Peking University, Beijing, People’s Republic of China

**Keywords:** Isolated maternal hypothyroxinaemia, Maternal outcomes, Offspring outcomes, Levothyroxine, Systematic review and meta-analysis

## Abstract

**Objective:**

To examine the association between isolated maternal hypothyroxinaemia (IMH) and adverse obstetric outcomes and offspring outcomes and also investigate the effects of levothyroxine therapy on IMH for the above outcomes.

**Methods:**

We systematically searched PubMed, EMBASE, and Cochrane Library, and the reference lists of key reviews were hand searched on June 9, 2021. Two authors independently screened titles/abstracts. Full articles were further assessed if the information suggested that the study met the inclusion/exclusion criteria, and two researchers performed data extraction and risk-of-bias assessment using standardized tables. Summary relative risks or the mean difference between maternal effects and offspring outcomes were calculated by a random-effects model.

**Results:**

We identified 38 eligible articles (35 cohort studies and two randomized controlled trials [RCT]). Meta-analysis showed that maternal IMH was associated with increased gestational diabetes mellitus, preterm premature rupture of membranes, preterm birth, fetal distress, and macrosomia outcomes in IMH compared to euthyroid women, and the relative risks were 1.42 (1.03–1.96), 1.50 (1.05–2.14), 1.33 (1.15–1.55), 1.75 (1.16–2.65) and 1.62 (1.35–1.94), respectively. IMH was not associated with placenta previa, gestational hypertension, pre-eclampsia, intrauterine growth restriction, and offspring outcomes like birth weight, low birth weight infants, fetal macrosomia, neonatal intensive care, neonatal death, or fetal head circumference. In addition, we did not find an association between IMH and adverse offspring cognitive defects. Due to insufficient data for meta-analysis, it failed to pool the evidence of levothyroxine’s therapeutic effect on IMH and their offspring.

**Conclusions and relevance:**

IMH in pregnancy may relate to a few maternal and offspring outcomes. Moreover, there is currently no sufficient evidence that levothyroxine treatment during pregnancy reduces adverse maternal outcomes and disability in offspring. Further investigation to explore the beneficial effects of levothyroxine therapy is warranted.

**Supplementary Information:**

The online version contains supplementary material available at 10.1007/s40618-022-01967-4.

## Introduction

The thyroid hormone regulates metabolism, growth, and development in most human body tissues, and the hormone levels during pregnancy are crucial to fetal and neonatal neuropsychological development [1]. The fetus is entirely dependent on maternal thyroid hormones via the placenta in the first trimester since the fetal thyroid cannot secrete thyroid hormones before 12–14 weeks of gestation [2, 3]. Maternal thyroid dysfunction may lead to adverse fetal and neonatal outcomes, such as miscarriage, placenta abruption, pre-eclampsia, premature delivery, and even reduced offspring intelligence [4].

Defined by American and European Thyroid Associations’ Guidelines, isolated maternal hypothyroxinaemia (IMH) is with free thyroxine (FT4) concentration in the lower 2.5–5th percentile of the pregnancy-related reference range in conjunction with a normal maternal thyroid-stimulating hormone (TSH) concentration [5, 6]. Emerging evidence suggests that IMH during pregnancy has increased in recent years [7, 8]. The prevalence of IMH in the pregnant population ranges from 1.3 to 23.9%, depending on the study [9], with the most frequently reported percentage being 8–10% [10, 11]. The causes of as well as potential consequences of IMH have not yet been fully elucidated, and the current evidence does not adequately explain the reasons for IMH, which is speculated to be mainly caused by iodine deficiency [7, 12] and iron deficiency [13, 14].

Several studies have investigated the effects of IMH, and the results show conflicting evidence regarding adverse obstetric and neurodevelopmental outcomes. Recent studies have revealed that IMH in pregnancy is associated with unfavorable pregnancy outcomes, such as preterm delivery [8, 15], preterm premature rupture of the membranes [16], spontaneous abortion [17, 18], and gestational hypertension [19, 20], etc. Also, a couple of studies have suggested a similar point of view [19, 21, 22]. Beyond that, some other researchers hold different perspectives [23–27]. What is worth mentioning, according to a recent systematic review that included 19 cohorts, is suggested that IMH was not associated with gestational hypertension or pre-eclampsia [28]. Moreover, due to inconsistent research results and insufficient evidence for adverse pregnancy outcomes with increased IMH, levothyroxine (LT4) treatment is not recommended in China or America [5, 29]. However, LT4 replacement is only advocated for this condition in the first trimester of pregnancy in Europe [6]. So, the application of LT4 therapy during pregnancy remains controversial.

Our study aims to evaluate the association between IMH and pregnancy and offspring outcomes and further investigate the effects of LT4 supplementation on pregnant women with IMH and the offspring's outcomes through a systematic review and meta-analysis.

## Materials and methods

### Data sources and searches

To identify studies for inclusion, we conducted a systematic literature search for articles on the association of IMH with adverse maternal and offspring outcomes and the effect of LT4 treatment published from database inception to June 9, 2021, using PubMed, Embase, and Cochrane Library databases. Controlled vocabulary terms (e.g., MeSH term) were used for each concept and keyword synonyms for the search strategies (Supplementary Table S1). In addition, we also manually searched the references of included studies and previous systematic reviews to identify further relevant studies.

### Study selection

Studies were included if they met all of the following criteria: (1) IMH pregnant women with offspring or IMH maternal accepted LT4 therapy during pregnancy; (2) referring estimation of the maternal and offspring outcomes, including but not limited to preterm birth of premature rupture of membranes (PROM), placenta previa, gestational hypertension, pre-eclampsia, intrauterine growth restriction, and gestational diabetes mellitus (GDM), as well as offspring birth weight, number of low birth weight infants, total malformations, fetal macrosomia, fetal distress, neonatal intensive care, neonatal death, and fetal head circumference and cognitive outcome [intelligence quotient (IQ)]. Studies were excluded if they: (1) were published as an abstract, letter to the editor, case report, or review, (2) failed to provide sufficient data or information for analysis, and (3) duplicated studies.

Potential studies were independently assessed via titles, keywords, and abstracts for suitability by ZL, LZX, and YY. Full texts were referred to when information in the records was inadequate for determination. Records not meeting the inclusion criteria were excluded, and the remaining were examined thoroughly. Any disagreement was resolved by discussion with a third author (WSF and SYF). The article and the flow chart were developed based on the Preferred Reporting Items for Systematic Reviews and Meta-analyses (PRISMA) statement.

### Data extraction and quality assessment

Two investigators extracted data independently using a pre-designed extraction form modified following a pilot test and assessed each included study's risk of bias (ZL and LZX). The revised extraction form comprised four parts: general information, methodological quality, clinical characteristics, and outcomes. Precisely, the extraction form consists of the first author, year of publication, country, study design, patient characteristics, age, thyroid status reference values, thyroid status, thyroid hormone (TH) values (exposure for cohort analysis), and LT4 supplementation (intervention for RCTs), as well as maternal and offspring outcomes, which were listed in the ‘Study Selection’ part of this manuscript. The Newcastle–Ottawa Scale (NOS) for assessing the quality of observational studies and the Cochrane risk-of-bias tool was used to evaluate cohort studies’ quality and randomized trials, respectively. The studies from the same cohort will be assessed carefully, and then the suitable ones (published lately and with a larger sample size) will be selected for the quantitative combination of outcomes.

### Data synthesis and analysis

All analyses were performed with Review Manager 5.3, and all statistical tests were two-sided. All outcomes analyses were carried out using a two-step approach with random-effect models to pool estimates of the studies and assess heterogeneity across studies using the *I*^*2*^ statistic and 95% CIs. The study weight was calculated using the inverse variance method. The risk ratio (RR) or mean difference was used to measure the relative risk/ risk difference between the two groups in selected studies. The forest plot was used to display the results visually. Results were considered statistically significant if the *p* value was < 0.05. The power of each original study result was calculated if the result was not proven to be statistically significant (i.e., *p* > 0.05). Heterogeneity between studies was tested with Cochran’s *Q* test (*P* < 0.10 was considered significant heterogeneity) and the *I*^*2*^ statistic (values of 25, 50, and 75% were considered low, moderate, and high degrees of heterogeneity, respectively).

Details of the protocol for this systematic review are registered on PROSPERO and can be accessed at https://www.crd.york.ac.uk/prospero/display_record.php?ID=CRD42021278471.

### Role of the funding sources

This study was funded by the National Natural Science Foundation (81,922,016, 81,870,607), Peking University Medicine Fund of Fostering Young Scholars’ Scientific AND Technological Innovation (BMU2022PYB035), Fundamental Research Funds for the Central University, Key Clinical Projects of Peking University Third Hospital (BYSYZD2021030), and Shandong Provincial Natural Science Foundation (ZR2019JQ25, ZR2020ZD14) of China. The study’s funders played no role in the study design, data collection, data analysis, data interpretation, or report writing.

## Results

### Search results and the general characteristics of the included studies

**Fig. 1:**
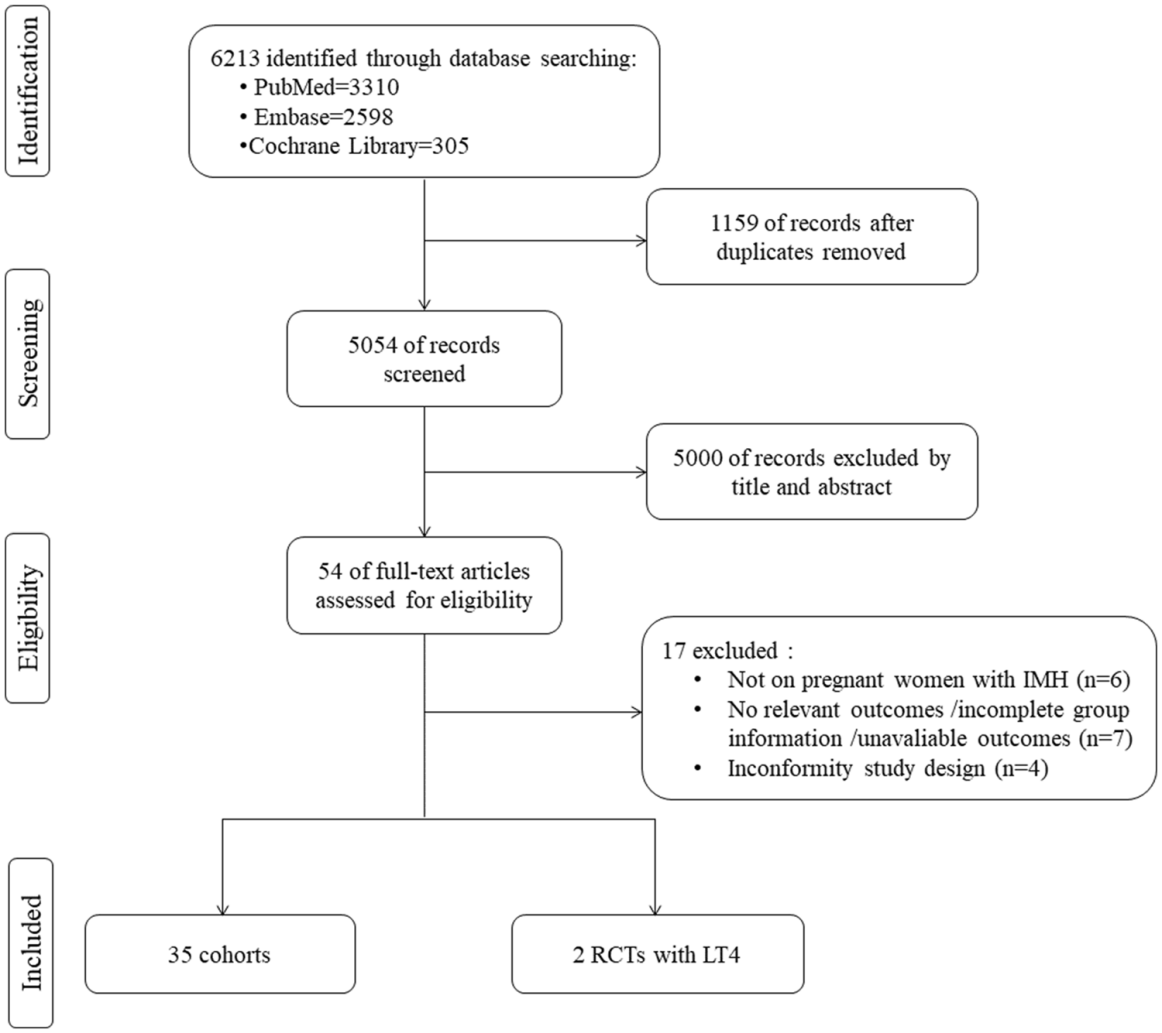
Study flow diagram for study selection

From 6213 published reports, 1159 were excluded after the duplicate deletion, and 54 remained eligible for inclusion based on the title and abstract screening. After reading the full text, a total of 35 cohorts and 2 RCTs matched the eligibility criteria (Fig. 1). The general characteristics of the 38 included studies published between 2003 and 2021 are presented in Table 1. Overall, the relevant studies were conducted in Netherlands (*n* = 12) [30–41] and China (*n* = 8) [8, 17, 19, 20, 42–45]. The geographic distribution of included studies can be found in Figure S1.

**Table 1:** (a) Cohorts of IMH meeting inclusion criteria, (b) RCTs of IMH meeting inclusion criteria

Study	Country	Source population	Population (IMH/euthyroid)	Gestational weeks at recruitment	Outcomes
(a)					
Pop [30]	Netherlands	Dutch Caucasian women in and around the city of Eindhoven	135 IMH/135 euthyroid	At 12 weeks gestation	The mental scale, the motor scale
Kooistra [31]	Netherlands	Dutch city of Veldhoven and its immediate vicinity	108 neonates born to women with IMH/96 euthyroid	At 12 weeks of pregnancy	Scores on the neonatal behavioral assessment scale orientation index
Casey [47]	America	Parkland Hospital	233 IMH/16,011 euthyroid	In the first half of pregnancy	Excessive adverse pregnancy outcomes
Cleary-Goldman [64]	America	FASTER trial funded by NICHD	10,990 patients (first trimester: 232 IMH/10,021 euthyroid; second trimester: 243 IMH/9981 euthyroid)	At first and second trimester	Preterm labor, macrosomia and gestational diabetes
Hamm [69]	Canada	Capital Health Region, Edmonton, Alberta	89 IMH/756 euthyroid	At 15–16 weeks of pregnancy	Risk for fetal growth restriction, preterm birth, or low Apgar score
Henrichs [32]	Netherlands	The Generation R Study	311 mild IMH and 157 severe IMH	At 13 ± 1.7 weeks	Children’s expressive vocabulary at 18 months, verbal and nonverbal cognitive functioning at 30 months
Li [42]	China	Shenyang Maternal and Neonatal Health Clinic	19 IMH/142 euthyroid	16–20 weeks	Intelligence scores and motor scores
Männistö [70]	Finland	NFBC 1986	227 IMH/4719 euthyroid	Population-based cohort, Northern Finland Birth Cohort 1986 (NFBC 1986)	Pregnancy complications or later maternal hypertension, diabetes, and thyroid disease
Su [17]	China	China-Anhui Birth Defects and Child Development cohort	43 IMH/845 euthyroid	At the first 20 weeks of pregnancy	Fetal distress, small for gestational age, and musculoskeletal malformations
Mil [33]	Netherlands	The Generation R Study	476 IH/4162 euthyroid	At a median gestational age of 13.4 weeks	Head circumference
Korevaar [15]	Netherlands	The Generation R Study	145 IH/4970 euthyroid	At 18 weeks gestation	Premature delivery, spontaneous premature delivery, very premature delivery
Finken [35]	Netherlands	ABCD study	175 IMH/1584 euthyroid	At a median gestational age of 90 days	Cognitive performance
Breathnach [71]	Ireland	NM	18 IMH/870 euthyroid	In the early second trimester	Placental abruption
Ghassabian [38]	Netherlands	The Generation R Study	3727 mother–child pairs and nonverbal intelligence quotient score at 6 years participated	At less than 18 weeks	Intelligence quotient score of the children
Román [36]	Netherlands	The Generation R Study	4039 children from mother-and-child cohort of the Generation R Study	Gestational weeks 6–18	Autistic symptoms in children
Medici [37]	Netherlands	The Generation R Study	5153 pregnant women	In early pregnancy	Mean blood pressures and hypertensive disorders, including pregnancy induced hypertension and preeclampsia
Ong [72]	Australia	Singleton pregnancies who attended WDP	244 IMH	Between 9 and 14 weeks gestation	Adverse pregnancy outcomes
Noten [39]	Netherlands	ABCD study	118 IH/1068 euthyroid	At a median gestational age of 12.9 weeks	Arithmetic performance
Modesto [40]	Netherlands	The Generation R Study	127 IMH/3560 euthyroid	13.6 ± 1.9 weeks	Children’s ADHD symptoms at 8 years
León [73]	Spain	The INMA	93 IH/1793 euthyroid	The first trimester of pregnancy	Birthweight, preterm delivery, gestational age (SGA/LGA)
Grau [74]	Spain	Catchment area of hospital	455 children (first trimester: 47 cases/39 euthyroid	At the end of first- and second trimester	Intellectual scores
Knight [10]	United kingdom	Exeter family study	82 IH/741 euthyroid	At 28 weeks gestation	Detailed anthropometric measurements (including BMI and skinfold thickness) and fasting blood samples (for TSH, free thyroxine (FT4), free triiodothyronine (FT3), HbA1c, lipid profile, plasma glucose and insulin resistance (HOMA-IR) analysis)
Furnica [21]	Belgium	NM	55 IH/165 euthyroid	At mean gestational of 11.8 weeks	BMI, fetal breech presentation, macrosomia and caesarian section rate
Oostenbroek [41]	Netherlands	ABCD study	200 hypothyroxinemia < 10th percentile/1800 non hypothyroxinemic	12.9 (interquartile range 11.9–14.1) weeks	Overall problem behavior, hyperactivity/inattention, conduct problems, emotional problems, peer relationship problems and prosocial behavior at age 5–6 years
Päkkilä [26]	Finland	NFBC 1986	71 IMH/4831 euthyroid	At 8–12 weeks gestation	Vision impairment
Nelson [25]	United Kingdom	The Avon Longitudinal Study of Parents and Children	93 IMH/4169 euthyroid	At median 10 weeks gestation	Children’s school performance or educational attainment
Zhu [44]	China	The MABC	78 IMH/3100 euthyroid	At Their first (mean = 10.01 weeks; SD 2.13) and second (mean = 25.64 weeks; SD 1.07)	SGA/LGA infants
Gong [20]	China	SHEP	89 IMH/756 euthyroid	In the early pregnancy	Macrosomia, gestational hypertension, gestational hypertension
Su [19]	China	IPMCH	342 with IMH/7831 unexposed		Pregnancy complications and neonatal outcomes
Yang [8]	China	IPMCH	963 IMH/40,948 euthyroid	At 9–13 weeks of pregnancy	Risk of preterm birth
Chen [43]	China	Obstetrical Department of the Third Hospital Affiliated to Wenzhou Medical University	70 IMH/1943 euthyroid	at 6–27 weeks of pregnancy	Placenta previa, placental abruption, fetal distress, fetal growth restriction, fetal distress, intrauterine fetal death, fetal malformation, premature rupture of membranes, premature birth, low birth weight infants, gestational diabetes mellitus and pregnancy-induced hypertension
Liu [45]	China	IPMCH	89 IMH/756 euthyroid	At 9–13 weeks of pregnancy	Macrosomia
Nazarpour [16]	Iran	TTPs	142 IMH/1701 euthyroid	NA	Preterm delivery, miscarriage, premature rupture of membrane (PROM), preterm premature rupture of the membranes (PPROM), low birth weight (LBW), third-trimester hemorrhage, neonatal admission, and the biometric neonatal parameters including birth weight (BW), birth height (BH) or birth head circumference (BHC)
Avramovska [75]	North Macedonia	Department of Gynecology and Obstetrics in Skopje	131 IMH/218 euthyroid	First trimester (up to 12 gestational weeks), second trimester (12–28 gestational weeks) and third trimester (28 gestational weeks to the end of pregnancy)	Adverse pregnancy outcome
Karbownik-Lewińska [76]	Poland	Department of Endocrinology and Metabolic Diseases, Medical University of Lodz	8 IH/240 euthyroid	Age range 13–57 years	Age, body mass, height, body mass index (BMI), red blood cells (RBC), hemoglobin (Hgb), white blood cells (WBC), neutrophils, lymphocytes, platelets, total cholesterol, HDL cholesterol (HDLC), LDL cholesterol (LDLC), HDLC/cholesterol ratio, triglycerides (TGs), glucose, iron concentration, vitamin D, insulin resistance index (IRI), and thyroid tests, including thyroid antibodies. IRI was calculated on the basis of glucose and insulin concentrations obtained during an oral glucose tolerance test (OGTT)
(b)					
Lazarus 2012 [49]	United kingdom	CATS	411 IMH (242 accept levothyroxine vs 169 no treatment)	Between 11 and 16 weeks	Cognitive function (full-scale IQ at 5 years of age, overall score from the Differential Ability Scales–II (DAS) at 3 years of age)
Casey 2017 [34]	America	NICHD Maternal Fetal Medicine Units Network	526 with IH (265 accept levothyroxine 50ug vs 261 placebo)	At a mean of 17.8 weeks	Intelligence quotient score

*IH* isolated hypothyroxinaemia, *IMH* isolated maternal hypothyroxinemia, *TSH* thyroid-stimulating hormone, *BMI* body mass index, *SGA/LGA* small/large-for-gestational-age, *IPMCH* international peace maternity and child health hospital, *ABCD study* Amsterdam born children and their development study, *FASTER trial* first and second-trimester evaluation of risk trial, *NICHD* the national institute of child health and human development, *NFBC 1986* Northern Finland Birth Cohort 1986, *NM*: not mentioned, *WDP* Western diagnostic pathology, *INMA* INfancia y Medio Ambiente (INMA-Environment and Childhood) study, *MABC* the Ma’anshan Birth Cohort, *TTPs* the Tehran Thyroid and Pregnancy study, *SHEP* subclinical hypothyroid in early pregnancy study

#### Included cohorts

In the cohort study part, the study population comprised 179,980 participants, of whom 8111 (4.36%) had IMH. After reading the full article thoroughly, we found multiple studies from the same cohort. First published in 2010, seven studies reported the source cohort as the Generation R Study, a prospective population-based cohort study from fetal life until young adulthood in a multi-ethnic urban population in the Netherlands [32–34, 36, 38, 40, 46]. In addition, three studies sourced from the ABCD cohort analyzed the association between IMH in early pregnancy and offspring cognitive outcomes [35, 39, 41]. Explicitly speaking, Pop (2003) [30], Kooistra (2006) [31], and Oostenbroek (2017) [41]were from the cohort established between 1997 and 1998 in Veldhoven, Netherlands. Additionally, it was also reported the source from NFBC 1986, China-Anhui Birth Defects and Child Development cohort, Exeter Family Study, FASTER trial funded by NICHD, SHEP, The Avon Longitudinal Study of Parents and Children, The INMA, the MABC, TTPs and electronic medical records from local hospitals.

Of the 35 cohorts, all of the studies included in the cohort analysis scored more than six stars, suggesting a low risk of bias (Table S2). All of the studies were with low risk on the representativeness of the exposed cohort, selection of nonexposed cohort, ascertainment of exposure, and outcome assessment. Around 77.14% (27/35) of the studies were at high risk of cohorts’ comparability based on the design or analysis. Most of the studies were fully and adequately followed up.

#### Included RCTs

In the two RCT studies, 937 pregnant women with IMH received LT4 supplementation compared throughout the pregnancy. Initially from two randomized controlled trials: (1) Randomized Trial of Thyroxine Therapy for Subclinical Hypothyroidism and Randomized Trial of Thyroxine Therapy and Hypothyroxinemia Diagnosed During Pregnancy, in which LT4 was compared with placebo [47, 48], and (2) in Controlled Antenatal Thyroid Screening (CATS), compared to no-treatment control [49]. No significant selection, performance, detection, attrition, reporting, or other biases were detected among the included articles (Figure S2A, S2B).

### Association between IMH and pregnancy /offspring outcomes

#### Pregnancy outcomes

**Fig. 2:**
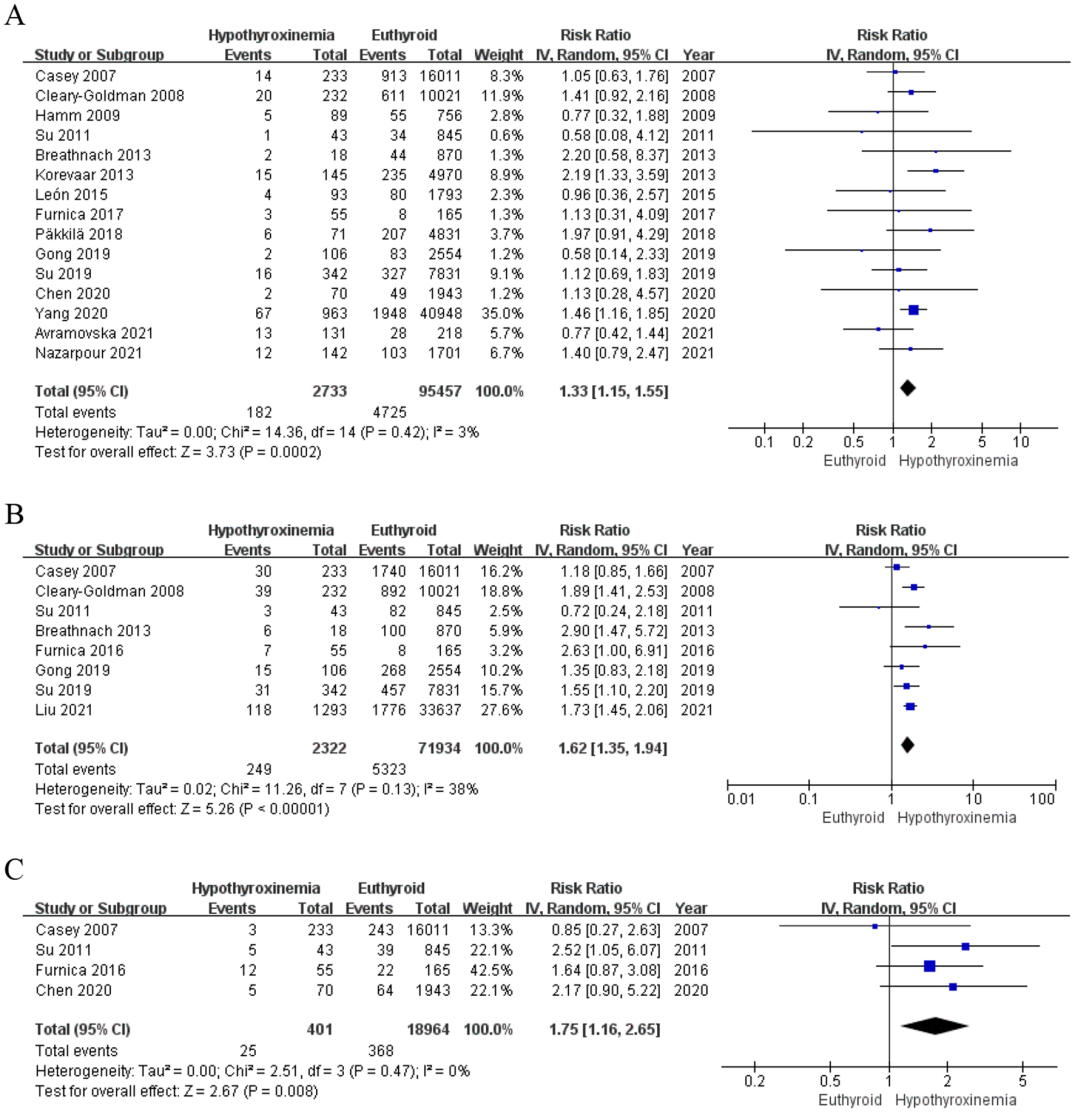
Forest plots of studies on the effect of IMH on the premature rupture of gestational diabetes mellitus, preterm premature rupture of membranes, preterm birth, fetal distress and macrosomia. **A** Forest plots of studies on the effect of IMH for preterm birth. **B** Forest plots of studies on the effect of IMH for total malformations. **C** Forest plots of studies on the effect of IMH for fetal distress

The association between IMH and pregnancy outcomes had wide variations in the amplitude of findings between studies included in this review. (1) In the 98,190 pregnancies from 15 cohorts, the risk of preterm birth was 6.66% (182 out of 2,733 IMH) vs 4.95% (4725 of 95,457 euthyroid women) in euthyroid women (RR [95% *CI*]; 1.33 [1.15, 1.55], *P* < 0.001), with a low heterogeneity (*I*^*2*^ = 3%, *p* = 0.42) (Fig. 2A). (2) In the PROM analysis, there was a statistically significant increase in PROM in the IMH group (RR [95% *CI*]; 1.50 [1.05, 2.14], *P* < 0.05), with no heterogeneity (*I*^*2*^ = 0%, *p* = 0.67) (Figure S3 A). (3) In the eight cohorts, that observed gestational diabetes, the meta-synthesis result showed that the IMH group had a higher risk of developing gestational diabetes (RR [95% *CI*]; 1.42 [1.03, 1.96], *P* < 0.05) (Figure S3 B). (4) In addition, in the nine studies that reported gestational hypertension as a maternal outcome, we found no association with IMH (RR [95% *CI*]; 1.21 [0.99–1.48]). Meanwhile, compared to euthyroid pregnancies, IMH did not significantly grow in placenta previa, pre-eclampsia, abruptio placentae, and intrauterine growth restriction of babies (Table 2 and Figure S3 C-G).

**Table 2:** The relationship between isolated maternal hypothyroxinemia and obstetric/infant/neonatal outcomes

Outcome type	Outcome	No. of studies	Hypothyroxinemia group		Euthyroid group		Risk ratio/Mean difference	Power
			Events	Total	Events	Total	Random, 95%CI	
Dichotomous	Preterm birth	15	182	2733	4725	95,457	1.33 (1.15–1.55)*	–
	Low Birth Weight Infants	9	54	1354	1734	41,289	0.93 (0.70–1.124)	0.053
	Fetal macrosomia	5	249	2322	5323	71,934	1.62 (1.35–1.94)*	–
	Premature rupture of membrane	7	30	1669	833	62,813	1.50 (1.05–2.14)*	–
	Abruptio Placentae	7	12	1228	239	43,949	2.56 (0.94–6.99)	0.458
	Small for gestational age	5	31	645	895	14,325	0.88 (0.53, 1.46)	0.267
	Fetal distress	4	25	401	368	18,964	1.75 (1.16–2.65)*	–
	Neonatal / fetal death	6	9	826	392	33,075	0.82 (0.45–1.50)	0.032
	Placenta previa	4	16	877	925	35,806	1.79 (0.64–5.01)	0.218
	Neonatal intensive care	4	19	448	527	18,747	0.88 (0.56–1.41)	0.401
	Total malformations	3	4	346	226	18,799	1.08 (0.36–3.19)	0.025
	Gestational diabetes mellitus	8	283	2191	7071	84,897	1.42 (1.03–1.96)*	–
	Intrauterine Growth Restriction	3	3	204	22	1253	0.54 (0.18–1.66)	0.029
	Gestational hypertension	9	104	2320	3820	89,348	1.21 (0.99–1.48)	0.075
	Pre-eclampsia	6	26	1273	1239	45,663	1.00 (0.67–1.48)	0.251
Continuous	Birth weights of offspring (g)	11	/	1796	/	22,849	28.45 (− 20.74, 77.64)	0.695
	Fetal head circumference (cm)	4	/	351	/	7750	− 0.01 (− 0.16, 0.15)	0.119
	Intelligence Score	3	/	195	/	3675	− 4.43 (− 11.35, 2.50)	0.999

Bold fonts indicate positive results, i.e., *P*<0.05

*P < 0.05

#### Fetal outcomes

Among 11 studies on birth weight outcomes, the studies Pop (2003) [30] and Kooistra (2006) [31] were from the same cohort, so Kooistra (2006) was included in the meta-analysis considering the sample size and published year. There was no significant difference in birth weight between babies born to IMH and euthyroid mothers (Table 2 and Figure S3 H). Similarly, the proportion of small for gestational age and low birth weight infants was no different between IMH and euthyroid mothers (Table 2 and Figure S3 I, J).

Within 8101 pregnancies, the fetal head circumference was 31.9 and 33.5 cm in IMH and euthyroid groups, respectively (*P* = 0.92) (Table 2 and Figure S3 K). In the three studies identified with intelligence evaluation, the IQ score of IMH offsprings was lower than euthyroid offsprings (105.4 vs 110, in IMH and euthyroid offsprings respectively; MD [95% CI]; − 4.43 [− 11.35, 2.50], *P* = 0.21). However, the means were not statistically different, and the heterogeneity between studies was 85% (Table 2 and Figure S3 L).

Of the meta-analysis in 8 cohorts, there was a statistically significant increase in macrosomic infants in the IMH group (RR [95% CI]; 1.62 [1.35, 1.94], *P* < 0.001) compared with the euthyroid group, with a moderate heterogeneity (*I*^*2*^ = 38%, *P* = 0.13) (Fig. 2B). Women with IMH had a higher risk of fetal distress babies vs euthyroid women (6.23 vs 1.94%, respectively; RR [95% CI]; 1.75 [1.16, 2.65], *P* < 0.05) (Fig. 2C). No differences were found in the rest of the analyses in neonatal intensive care and neonatal/fetal death outcomes between IMH and euthyroid women. (Table 2 and Figure S3 M–N).

#### Power analysis

In the power analysis, only two out of 18 (11.1%) outcomes (i.e., birth weights of offspring and intelligence score) achieved power over 0.5. No difference was found between the IMH and euthyroid group on the intelligence score outcomes, and the power was 0.999. Most of the rest of the outcomes obtained low to moderate power. Even the outcomes referring to gestational hypertension and pre-eclampsia only achieved a power lower than 0.10.

### The studies on the effect of LT4 supplementation on pregnancy outcomes/cognitive outcomes in individuals with isolated maternal hypothyroxinaemia

Based on the eligibility criteria, only two published RCTs were included for LT4 treatment in pregnant women with IMH. However, owing to the absence of further available randomized trials demonstrating the benefit of levothyroxine treatment for maternal hypothyroxinemia, screening for FT4 cannot be advocated.

Casey et al*. *[47] was secondary analyses of data from Randomized Trial of Thyroxine Therapy for Subclinical Hypothyroidism and Randomized Trial of Thyroxine Therapy for Hypothyroxinemia Diagnosed During Pregnancy at 15 centers within the Eunice Kennedy Shriver National Institute of Child Health and Human Development Maternal–Fetal Medicine Units Network. In Casey et al*. *[47], it was reported that 526 hypothyroxinaemia cases at 17.8 weeks were included. They found no significant differences in neurodevelopmental outcomes in children whose mothers had received LT4 treatment for IMH during pregnancy and the control groups (265/261) through a comprehensive battery of tests through 5 years of age. Using the same population data, Varner et al*. *[48] concluded no difference in neonatal TSH values between 461 newborns from pregnant women with IMH and those born to euthyroid women who were administered 50 $\mathrm{\mu g}$ of L-T4 supplementation during pregnancy from the same population, which was excluded as a duplicate study.

The result is consistent with the Controlled Antenatal Thyroid Screening (CATS) study conducted by Lazarus and his colleagues [49], which randomized 21 846 women recruited in early pregnancy (between 11 and 16 weeks gestation) to test TSH and FT4 levels during pregnancy versus serum sample storage and measurement after pregnancy. The subanalysis of the 411 mothers with IMH (defined as FT4 levels < P 2.5) found no significant improvement in cognitive function in children. Based on the current evidence, pregnancy outcomes and neonatal outcomes might not differ significantly between IMH with LT4 treatment and those without LT4 therapy during pregnancy. However, further studies are needed to validate these findings.

## Discussion

The effects of IMH on pregnancy and offspring outcomes are still controversial. This study performed a systematic review and meta-analysis to examine the differences in IMH and euthyroidism in pregnancy. Outcomes related to maternal health and neonatals’/offsprings’ growth and development were also summarized. Furthermore, we intended to verify the effects of LT4 supplementation on pregnancy outcomes and cognitive outcomes in children born to mothers with IMH through this systematic review.

In this systematic review and meta-analysis, IMH was associated with a higher risk of preterm birth than euthyroidism (6.66 vs 4.95%; RR, 1.33 [1.15, 1.55], *P* < 0.001). This finding was similar to a previous meta-analysis that included 47,045 pregnant women (containing 904 isolated hypothyroxinemia) individual data [15], which reported that among IMH women, the risk of preterm birth was 7.1 vs 5.0% in euthyroid women (OR, 1.46 [95% CI, 1.12–1.90]). IMH during pregnancy was also associated with a higher risk of maternal PROM, gestational diabetes, fetal macrosomia, and fetal distress compared with euthyroidism. Furthermore, there was no association between IMH and any other studied outcomes. Similar to previous studies [28], IMH was not associated with gestational hypertension. The above effects convincingly replicate the results of previous observational studies and systematic reviews.

The precise etiology of IMH is not fully understood. The iodine deficiency was recognized as a major high-risk factor of IMH because pregnant women with iodine deficiency had a significantly higher prevalence of hypothyroxinemia than those without [7, 12], and iodine deficiency would probably lead to a prior production of T3 to T4. Furthermore, some studies [13, 50, 51], reported that iron deficiency was another potential risk factor for IMH because the insufficient supply of iron deficiency in pregnant women had a significantly higher prevalence of hypothyroxinemia than those without. Some previous studies [52–54] proved that iron deficiency has multiple affects on the thyroid axis and was of crucial importance for thyroid hormone synthesis by reducing the activity of thyroid peroxidase. What’s more, environmental pollutants were found to be a significant inverse association with FT4 levels and no significant association with FT4 levels [55]. Besides these, including overweight/obesity [13, 45], maternal age [13], angiogenic factors, and thyroid peroxidase antibodies (TPOAb) [11, 20], were also reported as independent risk factors for IMH.

However, the mechanisms that cause this change and how to eliminate these risk factors remain unclear. Thyroid hormones regulate metabolism procedures and energy homeostasis [56]. Maternal thyroid hormones are essential for average physical growth and neurocognitive development from conception to adulthood [57]. Pop et al. [58] and Haddow et al. [59] reported that FT4 negatively correlates with body weight during pregnancy. Moreover, it has been confirmed that thyroid hormone therapy can improve blood glucose and insulin sensitivity in animal models [60]. The current study provides evidence that there was no consensus on the adverse effects of IMH on maternal–fetal outcomes, except for preterm birth, PROM, fetal macrosomia, and fetal distress. Moreover, we did not find many positive results, possibly because the conversion of T4 to T3 plays a role in ensuring a sufficient amount of T3. It is believed that lower FT4 levels could be compensated by higher peripheral deiodinase activity, resulting in a higher conversion rate of FT4 into active thyroid hormone FT3 and a higher FT3/FT4 ratio [59]. Those might lead to increased placental nutrition transfer or metabolism of fatty acids to the fetus, contributing to fetal macrosomia in the latter part of gestation [61]. Besides, the thyroid hormone plays a vital role in fetal neurodevelopment, associated with maternal thyroid hormones in the late first or early second trimester [57, 62, 63]. The fetus’s thyroid gland develops at 12 weeks and functions around 18 to 20 weeks of gestation. Therefore, even a mild decrease in thyroid hormone during the critical period will adversely affect brain development.

However, we still observed some differences from the published results. In our study, IMH was not associated with a higher risk for offspring’s intelligence and achieved a power greater than 0.99. In a meta-analysis of individual participant data from 9036 mother–child pairs from three prospective population-based birth cohorts, [22] FT4 < 2.5th percentile was associated with a 3.9-point (95% CI, − 5.7 to − 2.2) lower nonverbal IQ and a 2.1-point (95% CI, − 4.0 to − 0.1) lower verbal IQ. A suggestive association of hypothyroxinemia with a greater risk of autistic traits was observed.

The conflicting results of the above studies on the effects of IMH can be partly explained by various sources of heterogeneity between the individual studies. First of all, there are substantial differences in the definition of IMH, including the FT4 lower-range cutoffs and the normal ranges for TSH and FT4 between studies, which may affect the interpretation of the study results. Furthermore, the timing of the diagnosis of IMH differed considerably, ranging from 10 to 13 weeks up until 20 weeks of pregnancy [20, 64]. As discussed above, this might be important, as the study by Cleary-Goldman et al. suggested the trimester-specific effects of IMH [64]. In addition, the differences in covariates and the degree of endpoint measurements between studies could not be ignored. For example, orientated from the Generation R Study, Mil [33] and his colleagues described the maternal education level and delivery times in detail; In the study of follow-up of more than 40,000 pregnant women [8], the authors categorized premature delivery in detail, such as preterm birth (birth before 37 weeks gestation), very early preterm birth (birth before 34 weeks gestation), preterm premature rupture of membranes (preterm birth with spontaneous rupture of the membranes at less than 37 weeks gestation and before the onset of contractions), spontaneous preterm birth with intact membranes (spontaneous preterm birth with intact membranes), and medically-induced preterm birth (preterm birth after labor induction or cesarean delivery for maternal or fetal indications). In comparison, most studies are not able to differentiate between subgroups. Lastly, as mentioned, there are substantial differences in sample sizes between studies, with many studies working with only a limited number of cases leading to underpowered analyses, while some studies were based on a large population [8, 30, 31, 45]. Besides, albeit small power for most negative outcomes is also noteworthy.

Still, there is no consensus that the correction of IMH in pregnant women with LT4 treatment is beneficial [5, 6]. It is reported that the efficacy of levothyroxine treatment in reducing obstetric risk may vary by medical history and prior risk factors as well as by the timing of commencement of therapy. Treatment for hypothyroxinaemia beginning between 8 and 20 weeks of gestation has no significantly better cognitive outcomes in children through 5 years of age than no treatment for those conditions [47]. Based on the previous evidence, the pregnancy and neonatal outcomes are not significantly different between IMH with LT4 therapy and those without LT4 treatment [20, 47]. In the current study, only two RCTs were included in LT4 supplementation in pregnant women with IMH, and they were not suitable for synthesizing the outcomes quantitatively. Furthermore, more RCTs and clinical studies, especially with larger sample sizes using population- and pregnancy-specific reference ranges of FT4 and TSH levels, are still required to provide evidence for the efficacy of LT4 therapy. What also deserves expecting is new related studies in preparation and progress [65].

To the best of our knowledge, well designed and conducted, the current study is a systematic review and meta-analysis that roundly evaluated the association between IMH and pregnancy and offspring outcomes, while previous studies focused on a limited number of outcomes. Most of the previously reviewed studies focused on the effects of IMH on mental development in early life. As some of these cohorts have continued follow-up of their participants over the years, they have also studied cognitive performance in later life. Our study found no significant differences in most pregnancy outcomes or cognitive function in offspring between pregnant women with IMH and individuals with euthyroid, except for gestational diabetes mellitus, PROM, preterm birth, fetal distress, and macrosomia. In addition, given the existing interventional data, IMH might not be routinely treated during pregnancy.

There are some limitations to our study. First, we failed to specify the pregnancy period for enrolled pregnant women strictly. The fetal thyroid does not secrete thyroid hormones before 12–14 weeks of gestation during the first trimester, which may risk pregnancy outcomes and child cognitive development and neurodevelopmental disorders [66, 67]. In euthyroid pregnant women, FT4 levels increase, and TSH levels decrease, regulated by the increased human chorionic gonadotropin (hCG) levels during early gestation. The pregnant women with IMH in this study were in different stages of pregnancy, which might have affected the results. Besides, few trials could be included to synthesize quantitatively for each parameter of fetal-maternal and neonatal outcomes, despite extensive database searches. Finally, there were some limitations in those RCT studies. In Casey et al. [68] study, pregnant women earlier than eight weeks of gestation were not enrolled since they might have an early miscarriage. In Varner et al. [48] study, the newborn TSH levels were detected soon after birth at only a single point in time, which may not reflect thyroid function either in utero or later in life. Furthermore, the newborn TSH levels cannot be compared with those whose parents had a biochemical abnormality with euthyroid controls.

## Conclusions

Taken together, isolated maternal hypothyroxinaemia may be associated with gestational diabetes mellitus, preterm premature rupture of membranes and preterm birth in maternal, and fetal distress and macrosomia in offspring. The results of the current study provide further insights into the potential risks of isolated maternal hypothyroxinemia that may help optimize clinical decision-making strategies. Meanwhile, large detailed, and sufficiently powered studies using consistent definitions for IMH and adverse pregnancy outcomes still be required to clarify further the exact effects of IMH on pregnancy and offspring complications and elucidate the underlying pathophysiology of IMH. At the same time, the need for a sufficiently powered, placebo-controlled RCT to treat IMH pregnancies is emphasized.

## Supplementary Information

Below is the link to the electronic supplementary material.

Supplementary file1 (DOCX 359 KB)

## Abbreviations

IMH: Isolated maternal hypothyroxinaemia
IH: Isolated hypothyroxinaemia
RCT: Randomized clinical trial
TSH: Thyroid-stimulating hormone
L-T4: Levothyroxine
FT4: Free thyroxine
PROM: Premature rupture of membranes
IQ: Intelligence quotient
NOS: The Newcastle–Ottawa Scale
TH: Thyroid hormone
RR: Risk ratio
BMI: Body mass index
FPG: Fasting blood glucose
HOMA-IR: Homeostasis model assessment-insulin resistance
GDM: Gestational diabetes mellitus
LBW: Low birth weight
HCG: Human chorionic gonadotropin
PTB: Preterm birth
MeSH: Medical subject headings
