# Supplementary material for: Obstetric and offspring outcomes in isolated maternal hypothyroxinaemia: a systematic review and meta-analysis
Source: J Endocrinol Invest. 2022 Nov 23;46(6):1087–101. doi: 10.1007/s40618-022-01967-4 (PMC10185648; doi:10.1007/s40618-022-01967-4)
Supplement: Supplementary file 1 — Supplementary file1 (DOCX 359 KB) [file 40618_2022_1967_MOESM1_ESM.docx]

Table S1: Full search terms for MEDLINE (via Pubmed)

| Search number | Query | Search Details |
| --- | --- | --- |
| 17 | (((((((((((Thyroid Function Tests[MeSH Terms])) OR (Isolated maternal hypothyroxinemia)) OR (Isolated hypothyroxinemia)) OR (hypothyroxinemia)) OR (Thyrotroph Thyroid Hormone Sensitivity Index)) OR (Thyroid Gland Function Tests)) OR (Function Test, Thyroid)) OR (Test, Thyroid Function)) OR (Thyroid Function Test))) AND (((((Pregnant woman[MeSH Terms]) OR (Pregnancy[MeSH Terms])) OR (Pregnant Woman)) OR (Woman, Pregnant)) OR (Women, Pregnant)) | ("thyroid function tests"[MeSH Terms] OR (("isolate"[All Fields] OR "isolate s"[All Fields] OR "isolated"[All Fields] OR "isolates"[All Fields] OR "isolating"[All Fields] OR "isolation and purification"[MeSH Subheading] OR ("isolation"[All Fields] AND "purification"[All Fields]) OR "isolation and purification"[All Fields] OR "isolation"[All Fields] OR "isolations"[All Fields]) AND ("maternally"[All Fields] OR "maternities"[All Fields] OR "maternity"[All Fields] OR "mothers"[MeSH Terms] OR "mothers"[All Fields] OR "maternal"[All Fields]) AND ("hypothyroxinaemia"[All Fields] OR "hypothyroxinemia"[All Fields])) OR (("isolate"[All Fields] OR "isolate s"[All Fields] OR "isolated"[All Fields] OR "isolates"[All Fields] OR "isolating"[All Fields] OR "isolation and purification"[MeSH Subheading] OR ("isolation"[All Fields] AND "purification"[All Fields]) OR "isolation and purification"[All Fields] OR "isolation"[All Fields] OR "isolations"[All Fields]) AND ("hypothyroxinaemia"[All Fields] OR "hypothyroxinemia"[All Fields])) OR ("hypothyroxinaemia"[All Fields] OR "hypothyroxinemia"[All Fields]) OR ("thyroid function tests"[MeSH Terms] OR ("thyroid"[All Fields] AND "function"[All Fields] AND "tests"[All Fields]) OR "thyroid function tests"[All Fields] OR ("thyrotroph"[All Fields] AND "thyroid"[All Fields] AND "hormone"[All Fields] AND "sensitivity"[All Fields] AND "index"[All Fields]) OR "thyrotroph thyroid hormone sensitivity index"[All Fields]) OR ("thyroid function tests"[MeSH Terms] OR ("thyroid"[All Fields] AND "function"[All Fields] AND "tests"[All Fields]) OR "thyroid function tests"[All Fields] OR ("thyroid"[All Fields] AND "gland"[All Fields] AND "function"[All Fields] AND "tests"[All Fields]) OR "thyroid gland function tests"[All Fields]) OR ("thyroid function tests"[MeSH Terms] OR ("thyroid"[All Fields] AND "function"[All Fields] AND "tests"[All Fields]) OR "thyroid function tests"[All Fields] OR ("function"[All Fields] AND "test"[All Fields] AND "thyroid"[All Fields]) OR "function test thyroid"[All Fields]) OR ("thyroid function tests"[MeSH Terms] OR ("thyroid"[All Fields] AND "function"[All Fields] AND "tests"[All Fields]) OR "thyroid function tests"[All Fields] OR ("test"[All Fields] AND "thyroid"[All Fields] AND "function"[All Fields]) OR "test thyroid function"[All Fields]) OR ("thyroid function tests"[MeSH Terms] OR ("thyroid"[All Fields] AND "function"[All Fields] AND "tests"[All Fields]) OR "thyroid function tests"[All Fields] OR ("thyroid"[All Fields] AND "function"[All Fields] AND "test"[All Fields]) OR "thyroid function test"[All Fields])) AND ("pregnant women"[MeSH Terms] OR "pregnancy"[MeSH Terms] OR ("pregnant women"[MeSH Terms] OR ("pregnant"[All Fields] AND "women"[All Fields]) OR "pregnant women"[All Fields] OR ("pregnant"[All Fields] AND "woman"[All Fields]) OR "pregnant woman"[All Fields]) OR ("pregnant women"[MeSH Terms] OR ("pregnant"[All Fields] AND "women"[All Fields]) OR "pregnant women"[All Fields] OR ("woman"[All Fields] AND "pregnant"[All Fields]) OR "woman pregnant"[All Fields]) OR ("pregnant women"[MeSH Terms] OR ("pregnant"[All Fields] AND "women"[All Fields]) OR "pregnant women"[All Fields] OR ("women"[All Fields] AND "pregnant"[All Fields]) OR "women pregnant"[All Fields])) |
| 16 | ((((Pregnant woman[MeSH Terms]) OR (Pregnancy[MeSH Terms])) OR (Pregnant Woman)) OR (Woman, Pregnant)) OR (Women, Pregnant) | "pregnant women"[MeSH Terms] OR "pregnancy"[MeSH Terms] OR ("pregnant women"[MeSH Terms] OR ("pregnant"[All Fields] AND "women"[All Fields]) OR "pregnant women"[All Fields] OR ("pregnant"[All Fields] AND "woman"[All Fields]) OR "pregnant woman"[All Fields]) OR ("pregnant women"[MeSH Terms] OR ("pregnant"[All Fields] AND "women"[All Fields]) OR "pregnant women"[All Fields] OR ("woman"[All Fields] AND "pregnant"[All Fields]) OR "woman pregnant"[All Fields]) OR ("pregnant women"[MeSH Terms] OR ("pregnant"[All Fields] AND "women"[All Fields]) OR "pregnant women"[All Fields] OR ("women"[All Fields] AND "pregnant"[All Fields]) OR "women pregnant"[All Fields]) |
| 15 | ((((((((((Thyroid Function Tests[MeSH Terms])) OR (Isolated maternal hypothyroxinemia)) OR (Isolated hypothyroxinemia)) OR (hypothyroxinemia)) OR (Thyrotroph Thyroid Hormone Sensitivity Index)) OR (Thyroid Gland Function Tests)) OR (Function Test, Thyroid)) OR (Test, Thyroid Function)) OR (Thyroid Function Test)) | "thyroid function tests"[MeSH Terms] OR (("isolate"[All Fields] OR "isolate s"[All Fields] OR "isolated"[All Fields] OR "isolates"[All Fields] OR "isolating"[All Fields] OR "isolation and purification"[MeSH Subheading] OR ("isolation"[All Fields] AND "purification"[All Fields]) OR "isolation and purification"[All Fields] OR "isolation"[All Fields] OR "isolations"[All Fields]) AND ("maternally"[All Fields] OR "maternities"[All Fields] OR "maternity"[All Fields] OR "mothers"[MeSH Terms] OR "mothers"[All Fields] OR "maternal"[All Fields]) AND ("hypothyroxinaemia"[All Fields] OR "hypothyroxinemia"[All Fields])) OR (("isolate"[All Fields] OR "isolate s"[All Fields] OR "isolated"[All Fields] OR "isolates"[All Fields] OR "isolating"[All Fields] OR "isolation and purification"[MeSH Subheading] OR ("isolation"[All Fields] AND "purification"[All Fields]) OR "isolation and purification"[All Fields] OR "isolation"[All Fields] OR "isolations"[All Fields]) AND ("hypothyroxinaemia"[All Fields] OR "hypothyroxinemia"[All Fields])) OR ("hypothyroxinaemia"[All Fields] OR "hypothyroxinemia"[All Fields]) OR ("thyroid function tests"[MeSH Terms] OR ("thyroid"[All Fields] AND "function"[All Fields] AND "tests"[All Fields]) OR "thyroid function tests"[All Fields] OR ("thyrotroph"[All Fields] AND "thyroid"[All Fields] AND "hormone"[All Fields] AND "sensitivity"[All Fields] AND "index"[All Fields]) OR "thyrotroph thyroid hormone sensitivity index"[All Fields]) OR ("thyroid function tests"[MeSH Terms] OR ("thyroid"[All Fields] AND "function"[All Fields] AND "tests"[All Fields]) OR "thyroid function tests"[All Fields] OR ("thyroid"[All Fields] AND "gland"[All Fields] AND "function"[All Fields] AND "tests"[All Fields]) OR "thyroid gland function tests"[All Fields]) OR ("thyroid function tests"[MeSH Terms] OR ("thyroid"[All Fields] AND "function"[All Fields] AND "tests"[All Fields]) OR "thyroid function tests"[All Fields] OR ("function"[All Fields] AND "test"[All Fields] AND "thyroid"[All Fields]) OR "function test thyroid"[All Fields]) OR ("thyroid function tests"[MeSH Terms] OR ("thyroid"[All Fields] AND "function"[All Fields] AND "tests"[All Fields]) OR "thyroid function tests"[All Fields] OR ("test"[All Fields] AND "thyroid"[All Fields] AND "function"[All Fields]) OR "test thyroid function"[All Fields]) OR ("thyroid function tests"[MeSH Terms] OR ("thyroid"[All Fields] AND "function"[All Fields] AND "tests"[All Fields]) OR "thyroid function tests"[All Fields] OR ("thyroid"[All Fields] AND "function"[All Fields] AND "test"[All Fields]) OR "thyroid function test"[All Fields]) |
| 14 | Women, Pregnant | "pregnant women"[MeSH Terms] OR ("pregnant"[All Fields] AND "women"[All Fields]) OR "pregnant women"[All Fields] OR ("women"[All Fields] AND "pregnant"[All Fields]) OR "women pregnant"[All Fields] |
| 13 | Woman, Pregnant | "pregnant women"[MeSH Terms] OR ("pregnant"[All Fields] AND "women"[All Fields]) OR "pregnant women"[All Fields] OR ("woman"[All Fields] AND "pregnant"[All Fields]) OR "woman pregnant"[All Fields] |
| 12 | Pregnant Woman | "pregnant women"[MeSH Terms] OR ("pregnant"[All Fields] AND "women"[All Fields]) OR "pregnant women"[All Fields] OR ("pregnant"[All Fields] AND "woman"[All Fields]) OR "pregnant woman"[All Fields] |
| 11 | Pregnancy[MeSH Terms] | "pregnancy"[MeSH Terms] |
| 10 | Pregnant woman[MeSH Terms] | "pregnant women"[MeSH Terms] |
| 9 | Thyroid Function Test | "thyroid function tests"[MeSH Terms] OR ("thyroid"[All Fields] AND "function"[All Fields] AND "tests"[All Fields]) OR "thyroid function tests"[All Fields] OR ("thyroid"[All Fields] AND "function"[All Fields] AND "test"[All Fields]) OR "thyroid function test"[All Fields] |
| 8 | Test, Thyroid Function | "thyroid function tests"[MeSH Terms] OR ("thyroid"[All Fields] AND "function"[All Fields] AND "tests"[All Fields]) OR "thyroid function tests"[All Fields] OR ("test"[All Fields] AND "thyroid"[All Fields] AND "function"[All Fields]) OR "test thyroid function"[All Fields] |
| 7 | Function Test, Thyroid | "thyroid function tests"[MeSH Terms] OR ("thyroid"[All Fields] AND "function"[All Fields] AND "tests"[All Fields]) OR "thyroid function tests"[All Fields] OR ("function"[All Fields] AND "test"[All Fields] AND "thyroid"[All Fields]) OR "function test thyroid"[All Fields] |
| 6 | Thyroid Gland Function Tests | "thyroid function tests"[MeSH Terms] OR ("thyroid"[All Fields] AND "function"[All Fields] AND "tests"[All Fields]) OR "thyroid function tests"[All Fields] OR ("thyroid"[All Fields] AND "gland"[All Fields] AND "function"[All Fields] AND "tests"[All Fields]) OR "thyroid gland function tests"[All Fields] |
| 5 | Thyrotroph Thyroid Hormone Sensitivity Index | "thyroid function tests"[MeSH Terms] OR ("thyroid"[All Fields] AND "function"[All Fields] AND "tests"[All Fields]) OR "thyroid function tests"[All Fields] OR ("thyrotroph"[All Fields] AND "thyroid"[All Fields] AND "hormone"[All Fields] AND "sensitivity"[All Fields] AND "index"[All Fields]) OR "thyrotroph thyroid hormone sensitivity index"[All Fields] |
| 4 | hypothyroxinemia | "hypothyroxinaemia"[All Fields] OR "hypothyroxinemia"[All Fields] |
| 3 | Isolated hypothyroxinemia | ("isolate"[All Fields] OR "isolate s"[All Fields] OR "isolated"[All Fields] OR "isolates"[All Fields] OR "isolating"[All Fields] OR "isolation and purification"[MeSH Subheading] OR ("isolation"[All Fields] AND "purification"[All Fields]) OR "isolation and purification"[All Fields] OR "isolation"[All Fields] OR "isolations"[All Fields]) AND ("hypothyroxinaemia"[All Fields] OR "hypothyroxinemia"[All Fields]) |
| 2 | Isolated maternal hypothyroxinemia | ("isolate"[All Fields] OR "isolate s"[All Fields] OR "isolated"[All Fields] OR "isolates"[All Fields] OR "isolating"[All Fields] OR "isolation and purification"[MeSH Subheading] OR ("isolation"[All Fields] AND "purification"[All Fields]) OR "isolation and purification"[All Fields] OR "isolation"[All Fields] OR "isolations"[All Fields]) AND ("maternally"[All Fields] OR "maternities"[All Fields] OR "maternity"[All Fields] OR "mothers"[MeSH Terms] OR "mothers"[All Fields] OR "maternal"[All Fields]) AND ("hypothyroxinaemia"[All Fields] OR "hypothyroxinemia"[All Fields]) |
| 1 | (Thyroid Function Tests[MeSH Terms]) | "thyroid function tests"[MeSH Terms] |

| **Section and Topic** | **Item #** | **Checklist item** | **Location where item is reported** |
| --- | --- | --- | --- |
| **TITLE** | | |  |
| Title | 1 | Identify the report as a systematic review. | 1 |
| **ABSTRACT** | | |  |
| Abstract | 2 | See the PRISMA 2020 for Abstracts checklist. | 1 |
| **INTRODUCTION** | | |  |
| Rationale | 3 | Describe the rationale for the review in the context of existing knowledge. | 3 |
| Objectives | 4 | Provide an explicit statement of the objective(s) or question(s) the review addresses. | 4 |
| **METHODS** | | |  |
| Eligibility criteria | 5 | Specify the inclusion and exclusion criteria for the review and how studies were grouped for the syntheses. | 5 |
| Information sources | 6 | Specify all databases, registers, websites, organisations, reference lists and other sources searched or consulted to identify studies. Specify the date when each source was last searched or consulted. | 4 |
| Search strategy | 7 | Present the full search strategies for all databases, registers and websites, including any filters and limits used. | 4 |
| Selection process | 8 | Specify the methods used to decide whether a study met the inclusion criteria of the review, including how many reviewers screened each record and each report retrieved, whether they worked independently, and if applicable, details of automation tools used in the process. | 5 |
| Data collection process | 9 | Specify the methods used to collect data from reports, including how many reviewers collected data from each report, whether they worked independently, any processes for obtaining or confirming data from study investigators, and if applicable, details of automation tools used in the process. | 6 |
| Data items | 10a | List and define all outcomes for which data were sought. Specify whether all results that were compatible with each outcome domain in each study were sought (e.g. for all measures, time points, analyses), and if not, the methods used to decide which results to collect. | 6 |
|  | 10b | List and define all other variables for which data were sought (e.g. participant and intervention characteristics, funding sources). Describe any assumptions made about any missing or unclear information. | NA |
| Study risk of bias assessment | 11 | Specify the methods used to assess risk of bias in the included studies, including details of the tool(s) used, how many reviewers assessed each study and whether they worked independently, and if applicable, details of automation tools used in the process. | 6 |
| Effect measures | 12 | Specify for each outcome the effect measure(s) (e.g. risk ratio, mean difference) used in the synthesis or presentation of results. | 6-7 |
| Synthesis methods | 13a | Describe the processes used to decide which studies were eligible for each synthesis (e.g. tabulating the study intervention characteristics and comparing against the planned groups for each synthesis (item #5)). | 7 |
|  | 13b | Describe any methods required to prepare the data for presentation or synthesis, such as handling of missing summary statistics, or data conversions. | NA |
|  | 13c | Describe any methods used to tabulate or visually display results of individual studies and syntheses. | 7 |
|  | 13d | Describe any methods used to synthesize results and provide a rationale for the choice(s). If meta-analysis was performed, describe the model(s), method(s) to identify the presence and extent of statistical heterogeneity, and software package(s) used. | 6-7 |
|  | 13e | Describe any methods used to explore possible causes of heterogeneity among study results (e.g. subgroup analysis, meta-regression). | NA |
|  | 13f | Describe any sensitivity analyses conducted to assess robustness of the synthesized results. | 7 |
| Reporting bias assessment | 14 | Describe any methods used to assess risk of bias due to missing results in a synthesis (arising from reporting biases). | 6 |
| Certainty assessment | 15 | Describe any methods used to assess certainty (or confidence) in the body of evidence for an outcome. | 7 |
| **RESULTS** | | |  |
| Study selection | 16a | Describe the results of the search and selection process, from the number of records identified in the search to the number of studies included in the review, ideally using a flow diagram. | 7 |
|  | 16b | Cite studies that might appear to meet the inclusion criteria, but which were excluded, and explain why they were excluded. | NA |
| Study characteristics | 17 | Cite each included study and present its characteristics. | Table 1 |
| Risk of bias in studies | 18 | Present assessments of risk of bias for each included study. | Supplementary material 1 and 2 |
| Results of individual studies | 19 | For all outcomes, present, for each study: (a) summary statistics for each group (where appropriate) and (b) an effect estimate and its precision (e.g. confidence/credible interval), ideally using structured tables or plots. | 8-11 and figure 2-4 |
| Results of syntheses | 20a | For each synthesis, briefly summarise the characteristics and risk of bias among contributing studies. | 8-11 and figure 2-4 |
|  | 20b | Present results of all statistical syntheses conducted. If meta-analysis was done, present for each the summary estimate and its precision (e.g. confidence/credible interval) and measures of statistical heterogeneity. If comparing groups, describe the direction of the effect. | 8-11 and figure 2-4 |
|  | 20c | Present results of all investigations of possible causes of heterogeneity among study results. | NA |
|  | 20d | Present results of all sensitivity analyses conducted to assess the robustness of the synthesized results. | NA |
| Reporting biases | 21 | Present assessments of risk of bias due to missing results (arising from reporting biases) for each synthesis assessed. | NA |
| Certainty of evidence | 22 | Present assessments of certainty (or confidence) in the body of evidence for each outcome assessed. | Table 3 |
| **DISCUSSION** | | |  |
| Discussion | 23a | Provide a general interpretation of the results in the context of other evidence. | 11-14 |
|  | 23b | Discuss any limitations of the evidence included in the review. | 14 |
|  | 23c | Discuss any limitations of the review processes used. | 14 |
|  | 23d | Discuss implications of the results for practice, policy, and future research. | 14 |
| **OTHER INFORMATION** | | |  |
| Registration and protocol | 24a | Provide registration information for the review, including register name and registration number, or state that the review was not registered. | NA |
|  | 24b | Indicate where the review protocol can be accessed, or state that a protocol was not prepared. | NA |
|  | 24c | Describe and explain any amendments to information provided at registration or in the protocol. | NA |
| Support | 25 | Describe sources of financial or non-financial support for the review, and the role of the funders or sponsors in the review. | 7 |
| Competing interests | 26 | Declare any competing interests of review authors. | 15 |
| Availability of data, code and other materials | 27 | Report which of the following are publicly available and where they can be found: template data collection forms; data extracted from included studies; data used for all analyses; analytic code; any other materials used in the review. | 16 |

*From:*  Page MJ, McKenzie JE, Bossuyt PM, Boutron I, Hoffmann TC, Mulrow CD, et al. The PRISMA 2020 statement: an updated guideline for reporting systematic reviews. BMJ 2021;372:n71. doi: 10.1136/bmj.n71


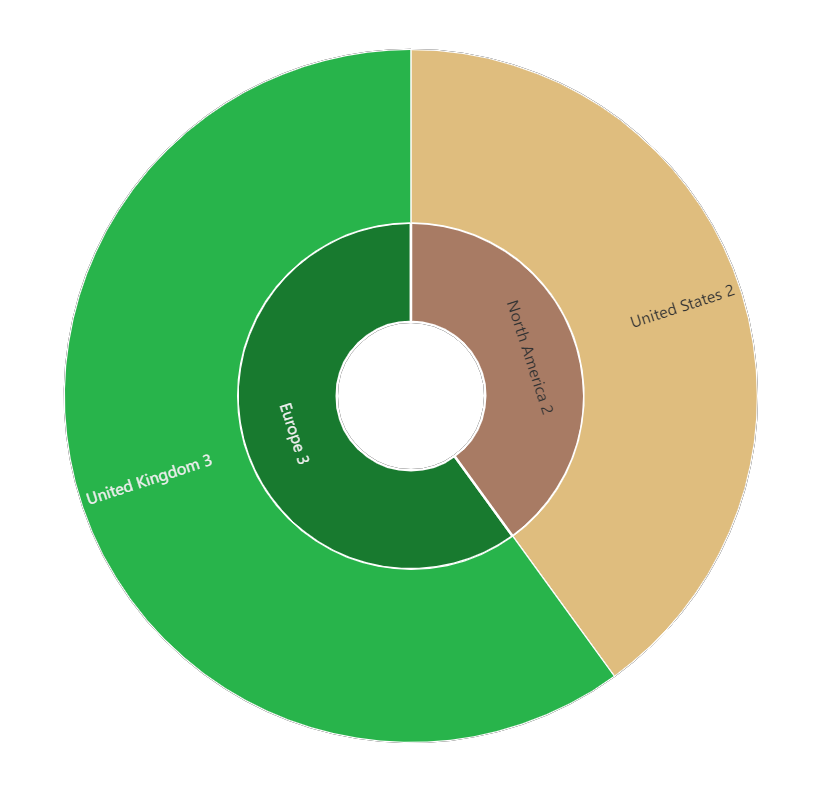

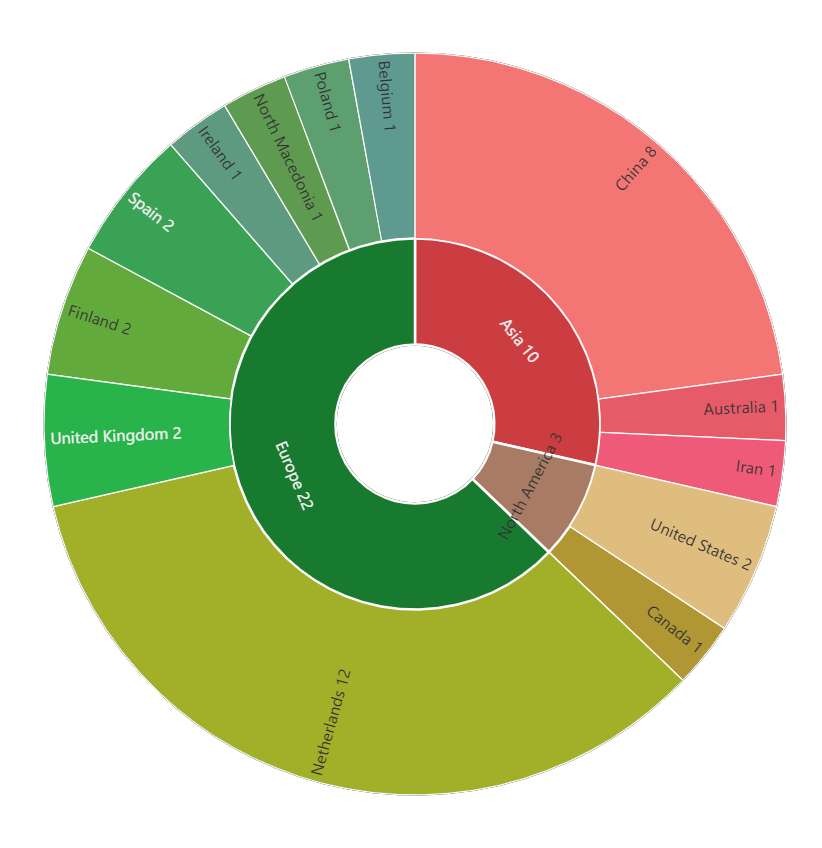
1a Distribution of included cohort studies 1b Distribution of included RCTs

Figure S1 Number of studies included in different countries

1. B.


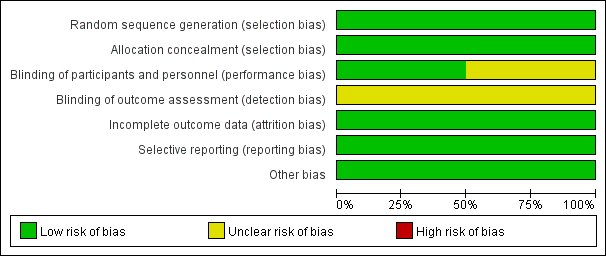

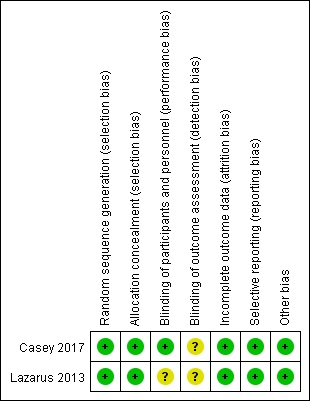


Figure S2 Risk of bias summary for three included RCTs


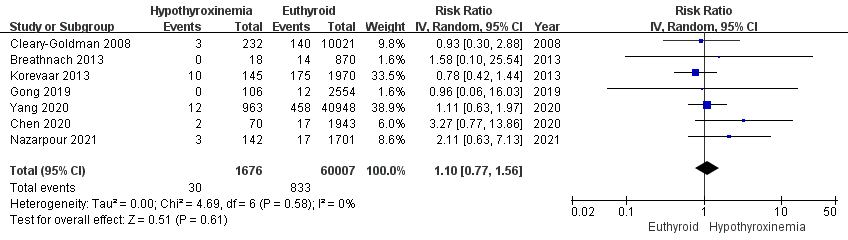


Figure S3 A. Forest plots of studies on the effect of IMH for PROM


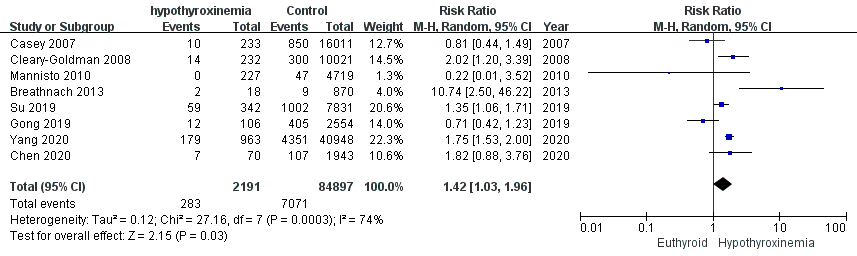


Figure S3 B. Forest plots of studies on the effect of IMH for gestational diabetes


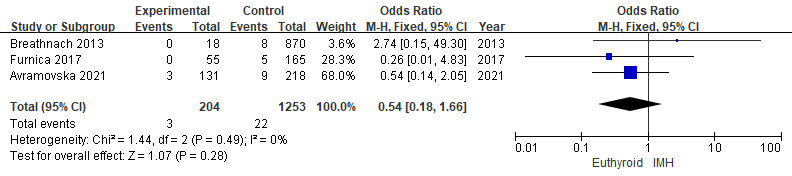


Figure S3 C. Forest plots of studies on the effect of IMH for Intrauterine Growth Restriction


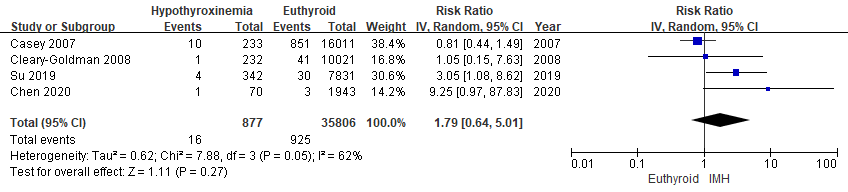


Figure S3 D. Forest plots of studies on the effect of IMH for Placenta previa


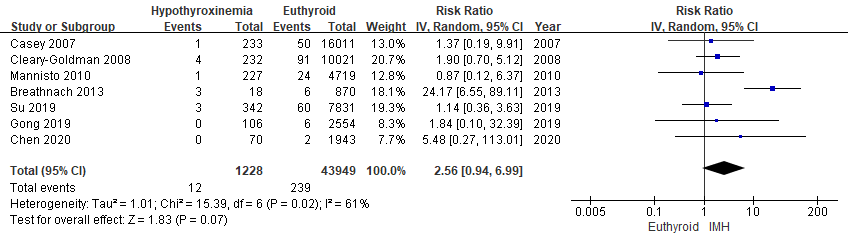


Figure S3 E. Forest plots of studies on the effect of IMH for Abruptio Placentae


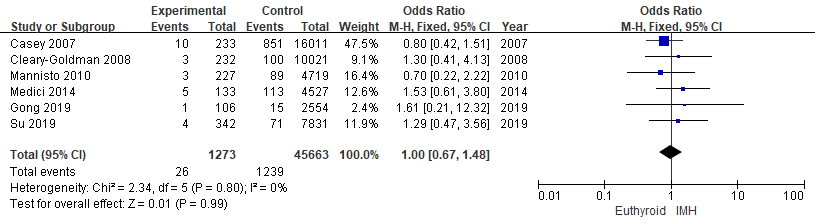


Figure S3 F. Forest plots of studies on the effect of IMH for Pre-eclampsia


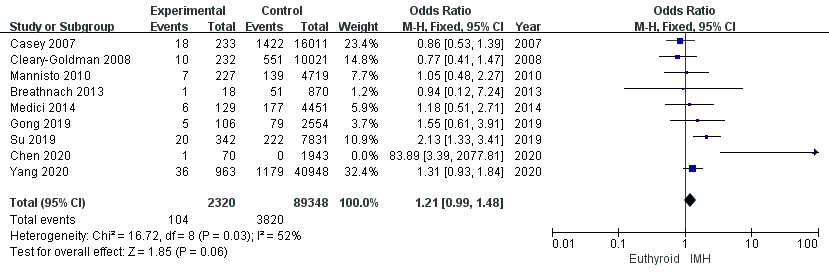


Figure S3 G. Forest plots of studies on the effect of IMH for Hypertension


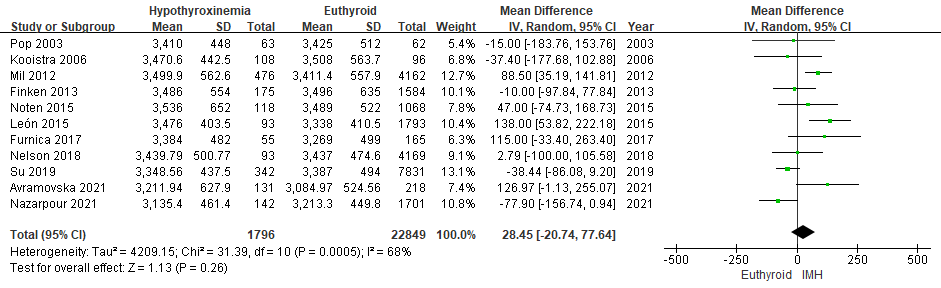


Figure S3 H. Forest plots of studies on the effect of IMH for Birth weights of offspring (g)


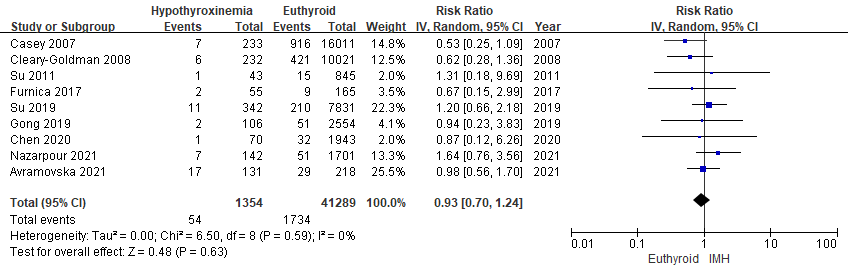


Figure S3 I. Forest plots of studies on the effect of IMH for Low Birth Weight Infants


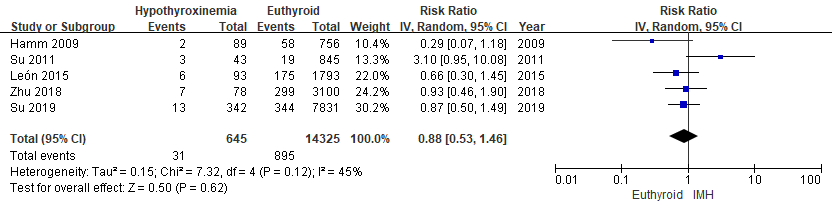


Figure S3 J. Forest plots of studies on the effect of IMH for Small for gestational age.


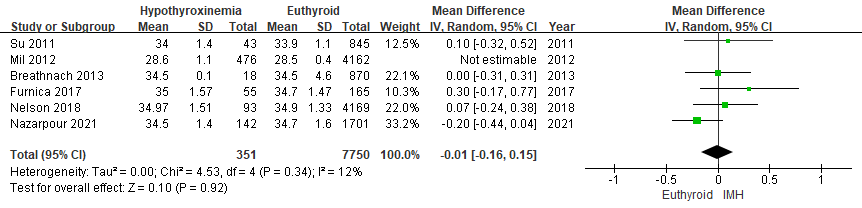


Figure S3 K. Forest plots of studies on the effect of IMH for Fetal head circumference (cm)


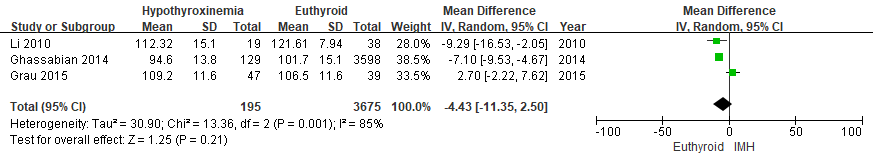


Figure S3 L. Forest plots of studies on the effect of IMH for Intelligence Score


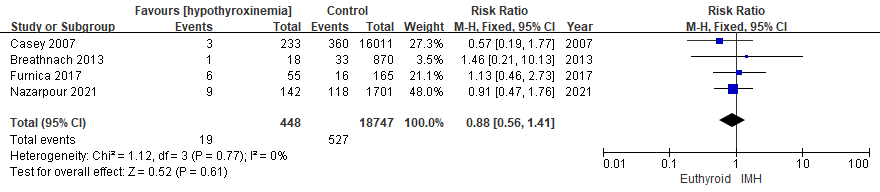


Figure S3 M. Forest plots of studies on the effect of IMH for Neonatal intensive care


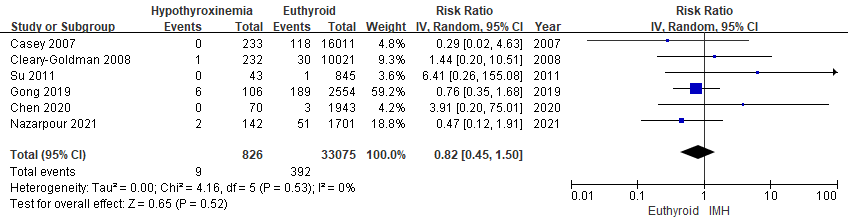


Figure S3 N. Forest plots of studies on the effect of IMH for Neonatal / Fetal death
